# Conserved residues within the HIV-1 Vpu transmembrane-proximal hinge region modulate BST2 binding and antagonism

**DOI:** 10.1186/s12977-017-0345-6

**Published:** 2017-03-14

**Authors:** Sabelo Lukhele, Éric A. Cohen

**Affiliations:** 1 Laboratory of Human Retrovirology, Institut de Recherches Cliniques de Montréal (IRCM), 110, Pine Avenue West, Montreal, QC H2W 1R7 Canada; 2 Division of Experimental Medicine, McGill University, Montreal, QC H3A 1A3 Canada; 3 Department of Microbiology, Infectiology and Immunology, Université de Montréal, Montreal, QC H3T 1J4 Canada

**Keywords:** HIV-1 Vpu, BST2 antagonism, Vpu–BST2 interaction, Vpu–BST2 trafficking, Vpu–CD4 interaction

## Abstract

**Background:**

BST2 inhibits HIV-1 release by tethering nascent virions to the surface of infected cells. HIV-1 Vpu overcomes this restriction by removing BST2 from viral budding sites via BST2 intracellular trapping and sequestration, surface downregulation and/or displacement mechanisms. Vpu is composed of a short luminal tail, a transmembrane domain (TMD) and a cytoplasmic hinge region that is followed by two helices. BST2 counteraction relies on the ability of Vpu to physically bind BST2 through TMD interactions and recruit the clathrin-dependent trafficking machinery via a canonical acidic di-leucine signalling motif within the helix-2 of Vpu. The highly conserved Vpu transmembrane-proximal hinge region encompasses residues that resemble an acidic leucine-based trafficking motif, whose functional roles are currently ill-defined. In this study, we investigated the contribution of these residues towards Vpu-mediated BST2 antagonism.

**Results:**

We show that while these conserved residues have no intrinsic activity on the cellular distribution of Vpu in the absence of BST2, they regulate the ability of Vpu to bind to BST2 and, consequently, govern both BST2-dependent trafficking properties of the protein as well as its co-localization with BST2. Moreover, these residues, particularly a glutamic acid residue positioned immediately following the TMD, are a determinant not only for efficient targeting of BST2, but also binding and degradation of CD4, another host membrane protein targeted by Vpu. Mechanistically, our data are consistent with a role of these residues in the maintenance of the Vpu TMD conformational configuration such that interactions with membrane-associated host targets are favoured.

**Conclusions:**

Altogether, this work demonstrates an important regulatory role of the transmembrane-proximal Vpu hinge region residues towards enabling the protein to efficiently engage its target host proteins. Thus, this highly conserved, cytosolic Vpu hinge region may represent an attractive target for the development of anti-Vpu inhibitors.

**Electronic supplementary material:**

The online version of this article (doi:10.1186/s12977-017-0345-6) contains supplementary material, which is available to authorized users.

## Background

BST2 (also referred to as Tetherin, CD317 or HM1.24) is a type I interferon (IFN-I)-induced membrane-associated restriction factor that inhibits HIV-1 release by directly cross-linking nascent virions to the surface of infected cells [1, 2]. Structurally, BST2 consists of a short N-terminal cytosolic domain, a transmembrane domain (TMD), followed by a coiled-coil ectodomain and a C-terminal glycosylphosphatidylinisotol (GPI) anchor. Tethering of virions results from insertion of one of the membrane anchors, preferentially the GPI anchor, into a budding virus particle while the other remains inserted into the host cell membrane [3]. In order to counteract BST2-mediated virion tethering, HIV-1 utilizes viral protein U (Vpu), a small membrane associated accessory protein that is also present in other related SIVs, but not in HIV-2 [4, 5]. Structurally, Vpu comprises a short luminal N-terminal tail, a TMD and a cytoplasmic domain that contains two helices separated by a linker region bearing two casein kinase II serine target sites. Phosphorylation of these serine residues (S52, S56) mediate recruitment of the β-TrCP2 subunit of the Skp1-Cullin1-F-Box (SCF^β-TrCP2^) E3 ubiquitin ligase [6, 7]. A short, flexible hinge region connects the TMD and the cytoplasmic domain. Maintenance of proper structural elements of Vpu, such as the TMD, is important for the ability of the protein to target BST2 as well as an array of other host factors, including CD4, NK-T-B-antigen (NTB-A), Polio virus receptor (PVR), sodium-coupled neutral amino acid transporter (SNAT1) and the C–C chemokine receptor-7 (CCR7) [8–13].

Vpu counteracts BST2 antiviral activity by mediating removal of BST2 from virus budding sites via intracellular trapping and sequestration, surface downregulation and/or displacement mechanisms [1, 2, 14–16]. A direct interaction between Vpu and BST2, which occurs via their respective TMDs, is critical for BST2 antagonism [9, 16, 17]. The interaction requires a Vpu A_10_xxxA_14_xxxA_18_xxxW_22_ hydrophobic TMD interface, as well as several other residues within the TMD [9, 16, 17]. This physical association, which is believed to occur in the endoplasmic reticulum (ER) and/or the *trans*-Golgi network (TGN), traps both newly synthesized and recycling BST2 within intracellular compartments including the TGN [14, 15, 18–20].

BST2 antagonism is closely linked to cellular distribution of Vpu, and is reliant on hijacking of the host clathrin-dependent trafficking machinery [18, 21, 22]. A canonical acidic di-leucine (ExxxLV) sorting motif within the second helix of Vpu from Clade B (VpuB) of the M group of HIV-1 (HIV-1M) facilitates recruitment of clathrin adaptor protein complexes (AP-1 and AP-2), leading to formation of ternary Vpu–BST2–AP complexes [16, 21, 22]. In addition to the sorting signal, recruitment of AP cofactors requires the interaction between Vpu and BST2, as well as phosphorylation of Vpu, both of which are believed to modulate conformational changes that promote interaction with AP complexes. Moreover, a dual tyrosine-based motif within the cytoplasmic tail of BST2 as well as the Vpu first helix, which forms non-canonical contacts with AP complexes, are also necessary [21, 22]. The Vpu di-leucine sorting motif targets BST2 to a yet-to-be-specifically defined endosomal compartment that is incompatible with transit of Vpu–BST2 complexes to the cell surface, and is also required for exclusion or lateral displacement of BST2 from viral assembly sites [16, 20, 23]. These actively sequestered Vpu–BST2 complexes are ultimately targeted for ESCRT-mediated endo-lysosomal degradation following BST2 polyubiquitination by SCF^β-TrCP2^ [24–26]. BST2 degradation, however, is generally dissociable from enhancement of virion release, especially in conditions of low BST2 expression levels [22].

Interestingly, even though the VpuB helix-2 di-leucine sorting signal drives the first step of BST2 counteraction, it is not conserved across all Vpu variants, as is most notable in Vpu variants from HIV-1M clades C (VpuC) and F. In these variants, curiously, an equivalent putative acidic di-leucine motif occurs within the transmembrane-proximal hinge region of the protein. Intriguingly, in addition to the VpuB helix-2 di-leucine signal, there exists a putative acidic di-leucine motif (ExxxIL) within the transmembrane-proximal hinge region. Therefore, based on the canonical sequence of acidic di-leucine motifs (D/ExxxLL/I/V/M) [27], VpuB has an optimal helix-2 signal while the hinge region putative motif would be predicted sub-optimal. Noteworthy, the membrane-proximal VpuB ExxxIL and membrane-distal ExxxLV mirror the major histocompatibility complex class II-associated invariant chain (CD74) membrane-proximal ExxxML and membrane-distal DxxxLI acidic di-leucine-based signals that are independently sufficient to recruit AP cofactors and target the transmembrane protein to endo-lysosomal compartments [28, 29].

Even though not optimal, there are positions within the VpuB transmembrane-proximal hinge region putative motif that are highly conserved, raising the possibility that the hinge region contributes to BST2 antagonism either by modulating Vpu trafficking, BST2 sequestration or through some other mechanism. To date, it is unclear whether this putative di-leucine motif plays any functional role. As well, the overall roles of the transmembrane-proximal hinge region within which the putative motif occurs are still ill-elucidated. As such, we set out to investigate the role of the VpuB hinge region residues, encompassing the putative sorting signal, towards Vpu-mediated BST2 antagonism. Additionally, considering the importance of the structural configuration of Vpu for targeting both BST2 and its other host protein target CD4, as well as the roles of disordered regions in modulating structural orientation or affording structural plasticity that facilitates interaction with target proteins, we further examined whether these conserved hinge region residues orient Vpu in such a way that binding to BST2 and CD4 is favoured.

In this study, we show that while the putative sorting motif within the transmembrane-proximal hinge region of Vpu does not influence the cellular localization of the protein in the absence of BST2, mutations of highly conserved residues within this region affect the subcellular distribution of Vpu and its co-localization with BST2 in BST2-expressing cells. We further show that these residues allow not only for optimal BST2 binding and counteraction but also for efficient CD4 interaction and degradation, suggesting a role of the transmembrane-proximal hinge region residues in properly positioning the Vpu TMD to efficiently engage its target host proteins. By demonstrating an important regulatory role of the transmembrane-proximal Vpu hinge region residues in BST2 binding, our study underscores the critical link between efficient binding of Vpu to BST2 and optimal trafficking of Vpu–BST2 complexes to endo-lysosomal compartments for sequestration and ultimately degradation.

## Results

### Highly conserved transmembrane-proximal hinge region residues influence Vpu subcellular distribution in a BST2-dependent manner

**Fig. 1:**
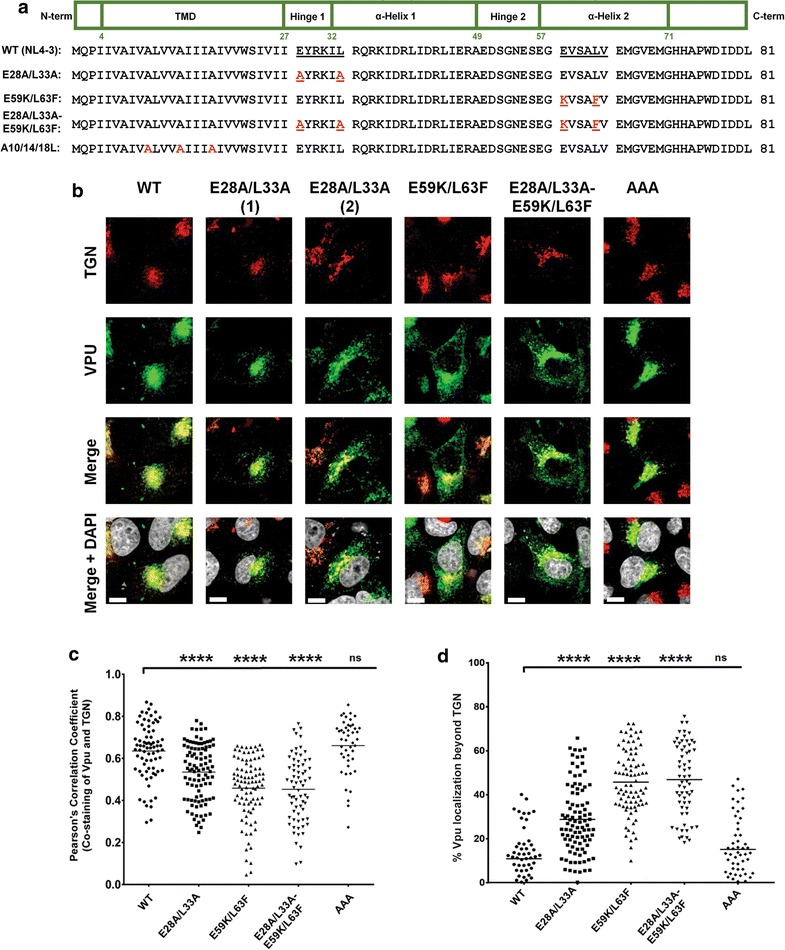
Membrane-proximal, hinge region E28/L33 residues are important for Vpu localization in the TGN. **a** Schematic representation of the structural domains and the sequence of the prototypical HIV-1 clade B NL4-3 Vpu (NL4-3, WT). Shown also are sequences of various Vpu mutants used in the study. **b** Intracellular localization of the Vpu mutants. HeLa cells were transfected with proviral plasmids encoding either WT Vpu or one of E28A/L33A, E59K/L63F, E28A/L33A-E59K/L63F and A10L/A14L/A18L Vpu mutants and were co-stained with anti-TGN46 (*red*, for TGN) and anti-Vpu (*green*) Abs as well as with DAPI (*grey*, for nucleus). Shown are representative confocal microscopy pictures for each of the Vpu mutants, with two prototypical patterns (1&2) of localization observed with the E28A/L33A mutant. **c, d** Quantification of the co-staining of anti-Vpu and anti-TGN46 Abs obtained from at least 50 distinct transfected cells per mutant. Shown are Pearson correlation coefficients (PCC) for each mutant (**c**) as well as Vpu distribution beyond the TGN (**d**). The percentage of Vpu distributing beyond the TGN was determined by calculating the ratio of the intensity of Vpu not co-staining with TGN versus total Vpu intensity in transfected cells. The *white bars* in B represent a distance of 10 µm and the *horizontal lines* (**c**, **d**) represent mean values of the PCC (**c**) and percentage of Vpu distributing beyond the TGN (**d**). Statistical analyses were performed using Mann–Whitney test

**Fig. 2:**
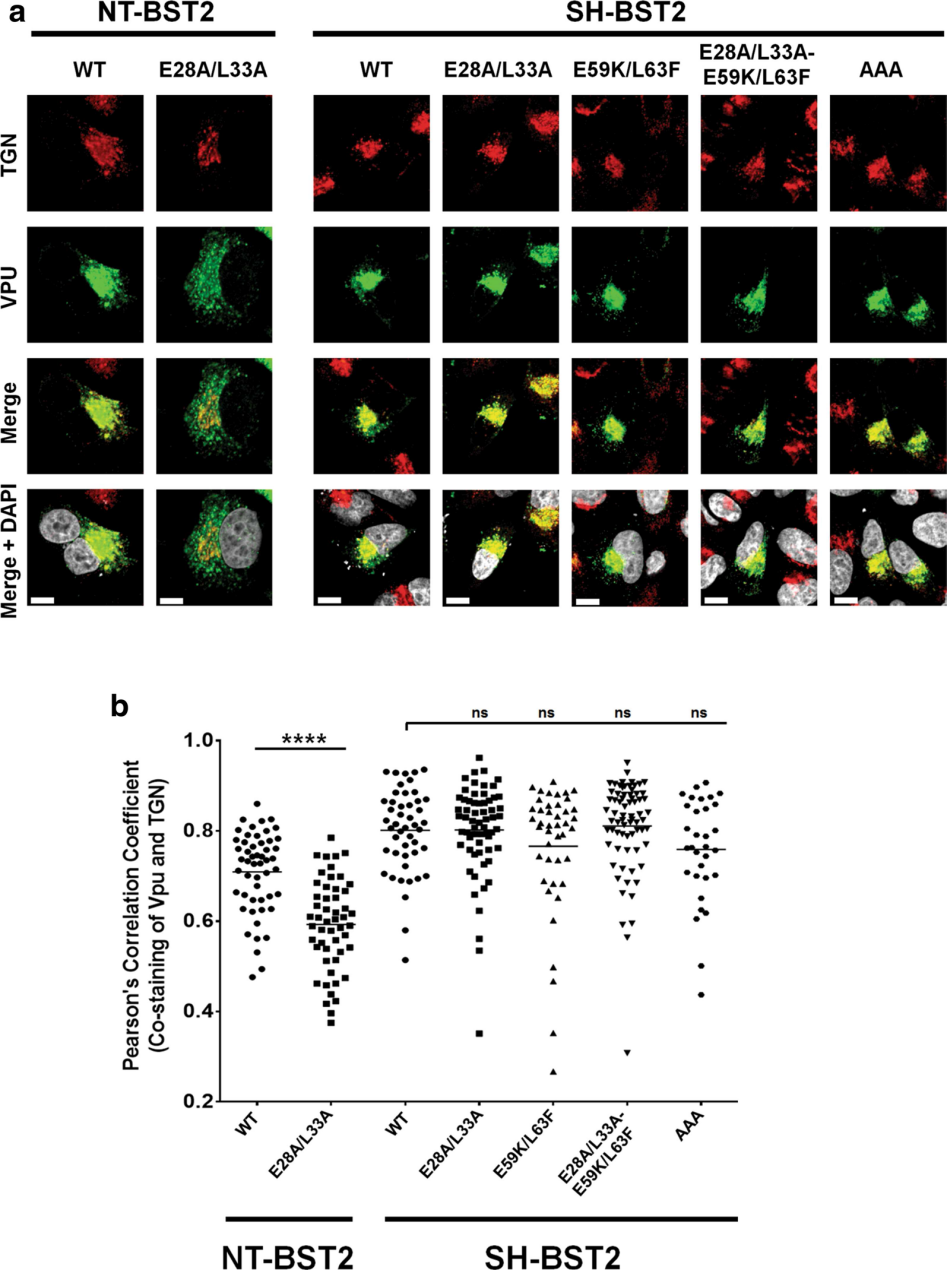
E28/L33 residues have no intrinsic activity on Vpu cellular distribution in the absence of BST2. **a** Intracellular localization of Vpu mutants in HeLa cells depleted of BST2 (SH-BST2, treated with shRNA against BST2) or not (NT-BST2, treated with non-targeting shRNA). Transfected cells were co-stained with anti-TGN46 (*red*, for TGN) and anti-Vpu (*green*) Abs as well as with DAPI (*grey*, for nucleus). Shown are representative confocal microscopy pictures for each of the Vpu mutants. The *white bars* represent a distance of 10 µm. **b** Quantification of the co-staining of anti-Vpu and anti-TGN46 Abs obtained from at least 50 distinct transfected cells per mutant. Shown are PCC values from each mutant. The *horizontal lines* represent the mean PCC. Statistical analyses were performed using Mann–Whitney test

In order to investigate the involvement of Vpu membrane-proximal hinge region residues (28**E**YRKI**L**33) in Vpu-mediated BST2 antagonism, we first evaluated their roles in governing Vpu cellular localization. Considering that in active di-leucine sorting motifs the glutamic acid (E) and leucine (L) residue positions are critical for activity, we generated provirus-based Vpu mutants that would allow for an assessment of whether the hinge region E28 and L33 residues modulate Vpu trafficking. These included mutants bearing mutations within the hinge region (28AYRKIA33, E28A/L33A), the second helix sorting signal (59KLSAFV63, E59K/L63F) while preserving the Env sequence, or both regions (E28A/L33A-E59K/L63F) (Fig. 1a). A Vpu A10L/A14L/A18L (Vpu-AAA) mutant, which is defective for BST2 interaction [9] was also included. Given the functional importance of Vpu accumulation within TGN compartments [14, 18, 20], we analysed the TGN distribution of the mutants in HeLa cells transfected with provirus plasmids expressing either wildtype (WT) Vpu or the various mutants. As previously reported [9, 18, 20], our confocal microscopy data revealed a preferential accumulation of WT Vpu in the TGN, as determined by the co-staining of Vpu with the TGN marker TGN46 (Fig. 1b–d). Importantly, the localization of the E28A/L33A mutant within the TGN was significantly reduced [Pearson correlation coefficient (PCC) = 0.54] relative to WT Vpu (PCC = 0.65) in these BST2-expressing cells. A corresponding two-fold increase in the percentage of Vpu distributing beyond the TGN was observed (16% for WT Vpu versus 35% for E28A/L33A) (Fig. 1d). Indeed, a redistribution of the mutant protein, albeit to varying extents, could be detected in punctuate structures beyond the TGN (see representative panels 1 and 2, Fig. 1b). An even greater reduction in TGN distribution was observed for both the E59K/L63F and E28A/L33A-E59K/L63F mutants (PCC = 0.43), in agreement with the increases in percentages of Vpu occurring beyond the TGN (52 and 54%, respectively) (Fig. 1b–d). Phenotypically, the E59K/L63F and E28A/L33A-E59K/L63F mutants redistributed towards the periphery in a comparable manner (Fig. 1b–d), indicating that mutation of the putative sorting motif in the hinge region did not augment the Vpu localization defect. Noteworthy, the accumulation of the Vpu-AAA mutant within TGN46-positive perinuclear compartments was comparable to WT Vpu (Fig. 1b–d). Of particular importance, none of the mutants showed any intrinsic defects in their TGN abundance following shRNA-mediated depletion of BST2 in HeLa cells (Fig. 2a, b and Additional file 1: Fig. S1), emphasizing that their differential cellular distribution is BST2-dependent. The fact that the BST2-binding defective Vpu mutant, Vpu-AAA, remains localized within the TGN as well as Vpu WT in the presence of BST2 (Fig. 1a, b) suggests that the Vpu redistributed outside the TGN likely represents Vpu whose trafficking properties are BST2-dependent.

**Fig. 3:**
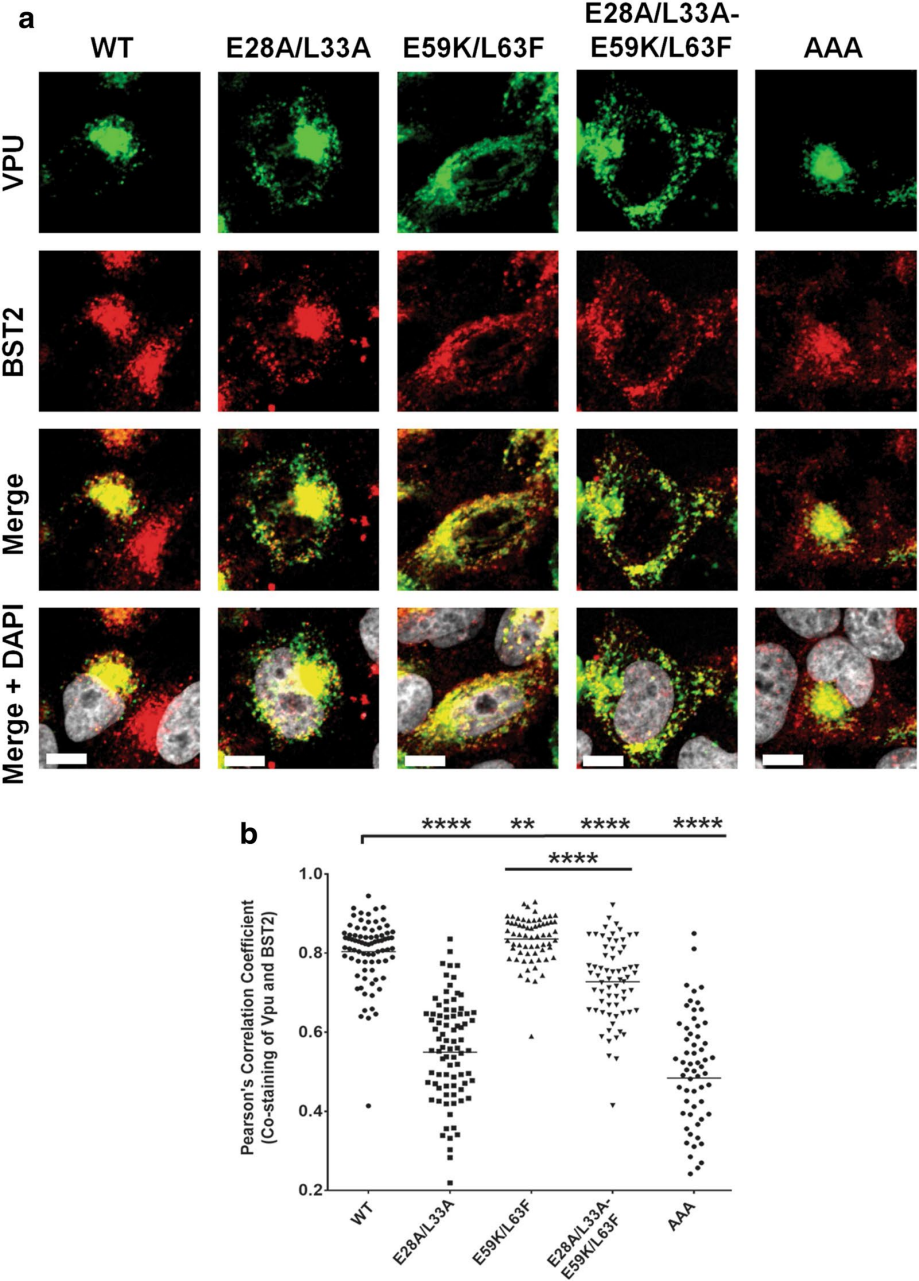
Hinge region E28/L33 residues are important for Vpu co-localization with BST2. **a** Representative pictures showing extent of co-localization of Vpu mutants with endogenous BST2 in HeLa cells as determined by the co-staining of anti-Vpu (*green*) and anti-BST2 (*red*) Abs following intracellular staining for both Vpu and BST2. **b** PCC values obtained from quantification of co-stainings of anti-Vpu and anti-BST2 Abs from at least 40 distinct transfected cells. The *white bars* in *panel* A represent a distance of 10 µm and the *horizontal lines* in *panel* B represent the mean PCC. Statistical analyses were performed using Mann–Whitney test

Given the BST2-dependent cellular distribution of the mutants, we next assessed the extent of their co-localization with BST2. Our data indicate that WT Vpu, which can efficiently bind, sequester and mediate degradation of BST2, co-localizes extensively with the restriction factor essentially within a perinuclear region. Interestingly, in the presence of the BST2 binding impaired Vpu-AAA mutant, BST2 subcellular distribution was altered with an increased localization outside the perinuclear region where co-localization with Vpu-AAA was minimal, highlighting that BST2 trafficking is influenced by formation of BST2 complexes (Fig. 3a, b). Given the reduced BST2 binding capacity of the Vpu-AAA mutant, its perinuclear co-localization with BST2 likely represents a mere overlap in staining resulting from the primary localization of both proteins in the TGN (Fig. 1a). Interestingly, the E28A/L33A mutant showed a statistically significant reduction in the extent of co-localization with BST2 compared to WT Vpu, in a large part because of a reduced co-localization outside the perinuclear region where most of the co-staining was detected (Fig. 3a, b). Relative to WT Vpu, the E59K/L63F mutant revealed an overall stronger co-localization with BST2 both outside and in a perinuclear region, most likely due to lack of degradation of Vpu–BST2 complexes in the case of this mutant (Additional file 1: Fig. S1). Importantly, the extent of BST2 co-localization of E28A/L33A-E59K/L63F was lower compared to the E59K/L63F mutant, even though both E59K/L63F and E28A/L33A-E59K/L63F do not mediate BST2 degradation (Additional file 1: Fig. S1; compare the levels of BST2 in the presence of Vpu E59K/L63F or E28A/L33A-E59K/L63F with those in the presence of the Vpu S52/56D mutant, which is unable to degrade BST2).

Taken together, our immuno-localization data indicate that the Vpu hinge region residues E28 and L33, which are part of a putative acidic di-leucine sorting motif, influence the BST2-dependent cellular distribution of Vpu beyond the TGN as well as the efficient co-localization of the protein with BST2. Moreover, the fact that mutations in the membrane-proximal hinge region motif did not augment the Vpu localization defect resulting from alterations of the second helix di-leucine motif, suggests a potential functional dependence between these two motifs.

### Vpu hinge region E28/L33 residues modulate the ability of Vpu to antagonize BST2

**Fig. 4:**
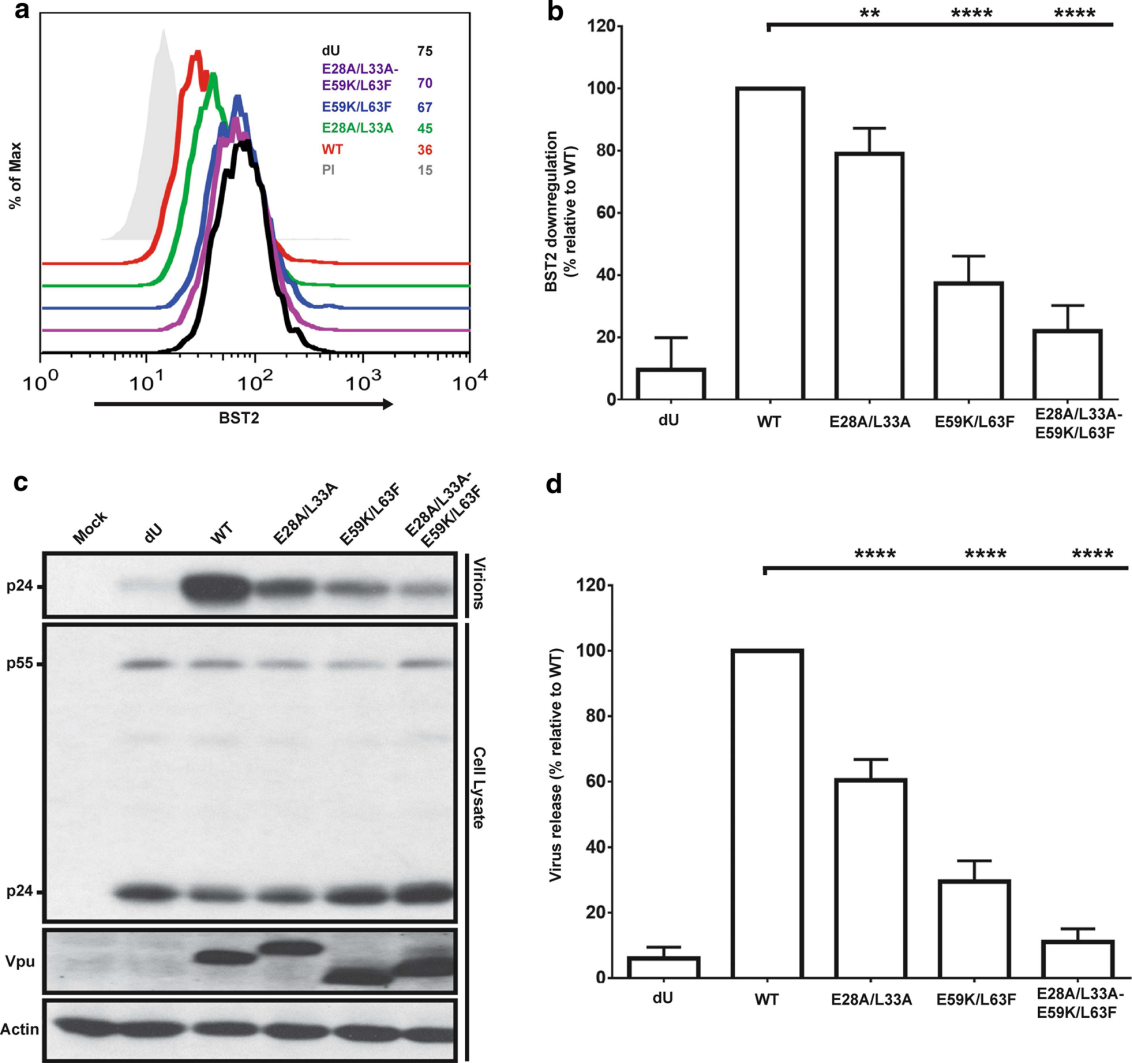
Conserved residues within the membrane-proximal hinge region of Vpu are important for BST2 counteraction. **a** A flow cytometry representative overlay showing the amount of surface BST2 following transfections of HeLa cells with either WT Vpu or the indicated Vpu mutants. Mean fluorescence intensity (MFI) values are indicated on the *right*. **b** A compilation of four independent experiments showing the extent of surface BST2 downregulation as determined by subtracting BST2 MFI values obtained from transfected cells (GFP+) from those in non-transfected cells (GFP−), and expressed as a percentage relative to the efficiency of BST2 downregulation obtained from WT Vpu, which in turn was arbitrarily set at 100%. **c**, **d** Efficiency of virus particle release following transfection of HeLa cells with provirus plasmids encoding WT Vpu and the indicated mutants. **c** A representative Western blot showing the amount of virion-associated p24 released into the supernatant (virion) and Gag products (p24 and p55) in the cell lysate. **d** A summary of quantifications of released virus particles by densitometric analyses of the intensity of Gag-related band signals from the Western blots from four different experiments. The efficiency of virus release was determined by calculating the ratio of virion-associated p24 released into the supernatant versus total Gag (cell- and virus-associated), and is expressed as a percentage of the release efficiency of the WT provirus, which in turn was set at 100%. For both (**b**) and (**d**), the *error bars* represent standard deviation (SD). Statistical analyses were performed using a two-way ANOVA, Tukey’s multiple comparison test

To test if the differential cellular distribution of the Vpu mutants has any functional consequences, we assessed their capabilities to downregulate surface BST2 as well as to enhance release of viral particles from transfected HeLa cells. Compared to WT provirus, a Vpu-deficient (dU) provirus was impaired in its surface BST2 downregulation function, as previously reported [2, 14, 24] (Fig. 4a). Meanwhile, the E28A/L33A mutant was moderately, albeit significantly, attenuated for BST2 downregulation (Fig. 4a, b). In line with earlier reports [20], the E59K/L63F mutation drastically reduced the extent of Vpu-mediated BST2 cell surface downregulation. Of particular note, simultaneously mutating both regions (E28A/L33A-E59K/L63F) yielded a cumulative defect on surface BST2 downregulation. In terms of BST2 antagonism, as measured by the ability of Vpu to enhance release of nascent virions, the dU mutant was unable to counteract BST2 (Fig. 4c, d). Most importantly, the ability of the E28A/L33A mutant to promote virus release was significantly attenuated. Consistent with the BST2 downregulation phenotype, the E59K/L63F mutant gave an even more pronounced attenuation. Yet again, the E28A/L33A-E59K/L63F mutant displayed a cumulative effect of the two mutated regions, giving a phenotype that is slightly higher to that of dU. It is worth mentioning that the E28A/L33A mutant was still able to mediate some BST2 degradation, albeit to a lesser extent than the WT Vpu (Additional file 1: Fig. S1), unlike the E59K/L63F, E28A/L33A-E59K/L63F and β-TrCP binding-deficient S52/56D mutants that were totally defective.

Altogether, our data highlight the significance of the conserved hinge region E28/L33 residues towards overcoming BST2 restriction. Of note, their contribution to BST2 antagonism is additive with that of the second helix di-leucine sorting signal, suggesting that they act at different stages of the counteraction.

### Vpu E28/L33 residues are important for binding to BST2

**Fig. 5:**
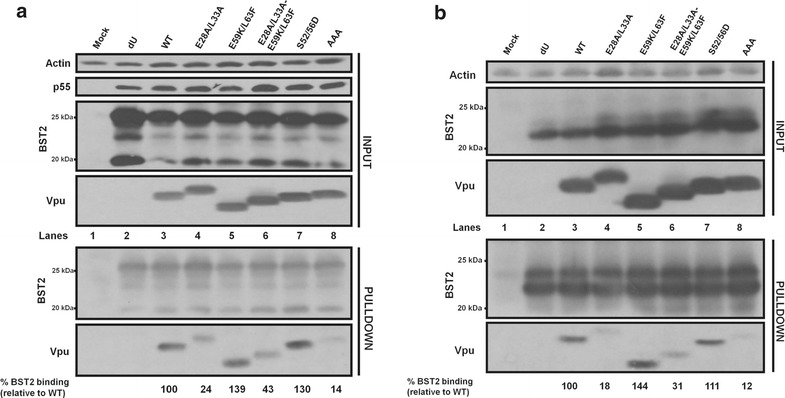
E28/L33 residues are important for binding of Vpu to BST2. **a** HEK293T cells were co-transfected with a proviral construct encoding WT Vpu or the indicated Vpu mutants and a BST2 expressor. Co-IP assays were then performed using an anti-BST2 Ab to pull down Vpu. Shown is a representative Western blot indicating the expression levels of proteins of interest in both the input lysate [actin (loading control), p55 (transfection control), BST2 and Vpu] and the immunoprecipitated fraction (BST2 and Vpu). **b** Co-IP following co-transfection of HEK293T cells with a proviral construct encoding WT Vpu or the indicated Vpu mutant and an expressor encoding for the short isoform of BST2 that contains the TMD involved in Vpu binding but is insensitive to Vpu-mediated degradation. Below each blot is the extent of BST2 binding of each Vpu mutant, relative to WT Vpu (set at 100%). For each condition, BST2 binding efficiency was determined from the ratio obtained from densitometric analyses of the intensities of Vpu- and BST2-related band signals in the immunoprecipitated fractions

**Fig. 6:**
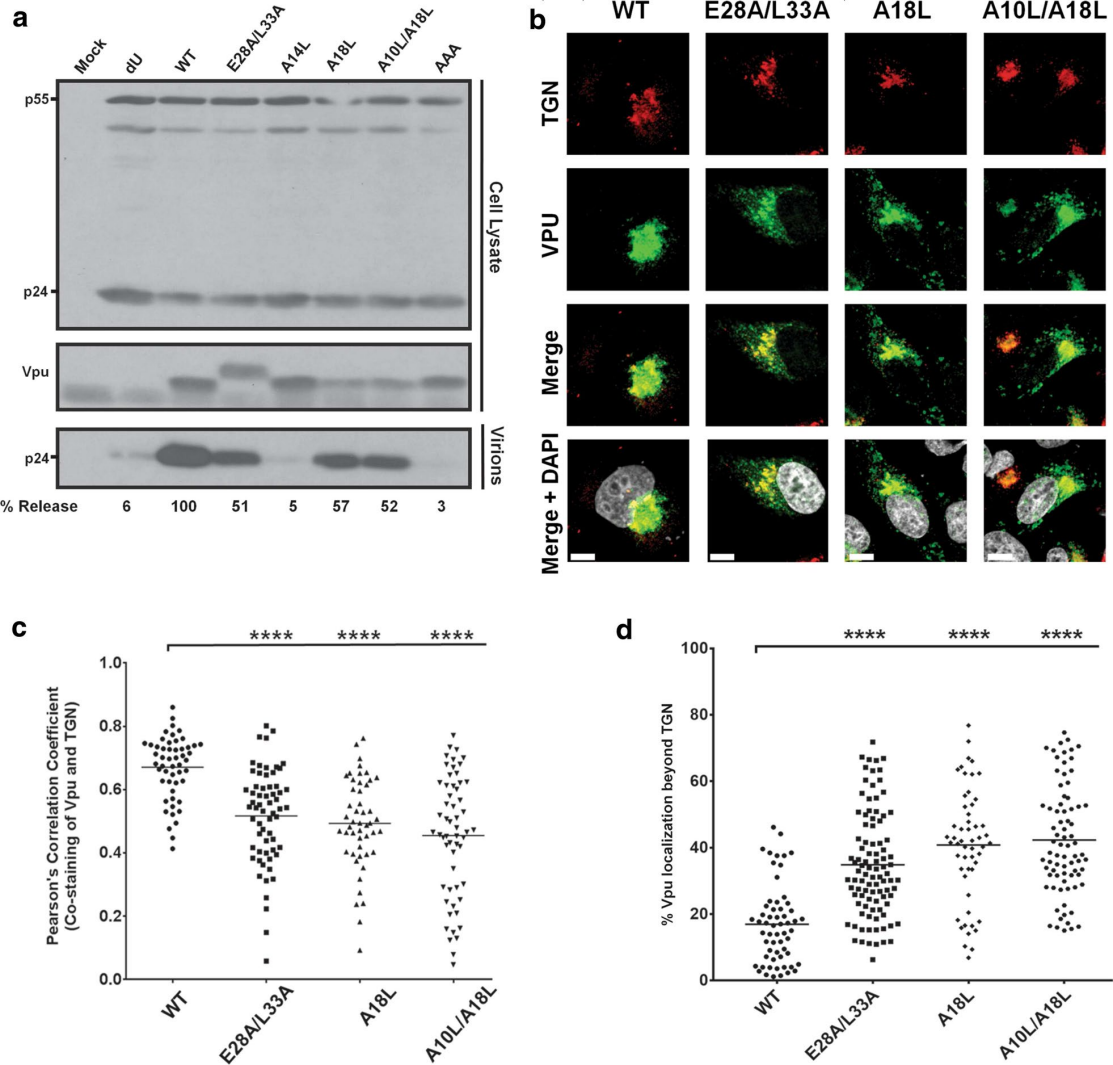
Impaired BST2-binding Vpu TMD mutants display defects in cellular distribution. **a** Enhancement of virus release by Vpu TMD and E28A/L33A mutants. A representative Western blot showing the amount of virion-associated p24 released into the supernatant (virion) and Gag products (p24 and p55) in the cell lysate. Below the blot are values indicating the efficiency of virus release (quantified and expressed as mentioned in Fig. 4 legend). **b**–**d** Vpu TMD mutants are defective for TGN localization. **b** HeLa cells transfected with either WT, E28AL/L33A, A18L or A10L/A18L Vpu mutants were co-stained with anti-TGN46 (*red*, for TGN) and anti-Vpu (*green*) Abs as well as with DAPI (*grey*, for nucleus). Shown are representative confocal microscopy pictures for each of the Vpu mutants. **c**, **d** Quantification of the co-staining of anti-Vpu and anti-TGN46 Abs obtained from at least 50 distinct transfected cells per mutant. Shown are Pearson correlation coefficients (PCC) for each mutant (**c**) as well as Vpu distribution beyond the TGN (**d**). The percentage of Vpu distributing beyond the TGN was determined by calculating the ratio of the intensity of Vpu not co-staining with TGN versus total Vpu intensity in transfected cells. The *white bars* in B represent a distance of 10 µm and the *horizontal lines* (**c**, **d**) represent mean values of the PCC (**c**) and percentage of Vpu distributing beyond the TGN (**d**). Statistical analyses were performed using Mann–Whitney test

Having shown the defect of these hinge region residues on BST2 antagonism and co-localization, we next evaluated the ability of the E28A/L33A mutant to interact with BST2. Towards this, we performed co-immunoprecipitation (Co-IP) assays in which we co-transfected proviral plasmids expressing the appropriate Vpu mutants together with a BST2 expressor into HEK293T cells, and pulled down Vpu using anti-BST2 antibodies (Abs). As shown in Fig. 5a, whereas WT Vpu and S52/56D were efficiently pulled down (lanes 3 and 7), there was no enrichment for a Vpu-AAA mutant known to be defective for BST2 interaction (lane 8), in accordance with the importance of the TMD for binding to BST2 [9]. Importantly, while the E59K/L63F mutant was efficiently pulled down (compared to the WT) in these assays (lanes 3 and 5), the E28A/L33A mutant was significantly attenuated for binding to BST2 (compare lane 4 with lane 3). A largely comparable binding defect was observed with the E28A/L33A-E59K/L63F mutant (compare lane 6 with lanes 3 and 5). The binding defect of the E28A/L33A mutant was independent of the degradation of the Vpu–BST2 complexes as it could not be rescued when the assay was performed using a BST2 expressor encoding for a short isoform of BST2 that contains the TMD involved in Vpu binding but is insensitive to Vpu-mediated downregulation and degradation (Fig. 5b) [30, 31]. Thus, our data indicate that the hinge region E28/L33 residues are important for the ability of Vpu to optimally interact with BST2, a condition that is necessary for Vpu-mediated BST2 counteraction. These results further suggest that the attenuated binding of E28A/L33A to BST2 could be responsible for the observed cellular localization and BST2 co-localization defects (Figs. 1, 3). In order to test this, we introduced mutations in Vpu TMD residues (A14L, A18L and A10L/A18L) that are relevant for binding to BST2 and correlated their binding strength (or lack thereof) to their cellular distribution. While the Vpu A14L was severely impaired for both BST2 binding and counteraction (resembling the Vpu-AAA mutant), the A18L and A10L/A18L gave intermediate BST2 binding and BST2 antagonistic phenotypes (Additional file 2: Fig S2 and Fig. 6a). In fact, their BST2 binding and functional profiles were comparable to the E28A/L33A mutant. Very importantly, the Vpu A18L and A10L/A18L mutants distributed beyond the TGN in a manner mirroring that obtained with the E28A/L33A mutant (Fig. 6b–d). Thus, the Vpu–BST2 binding affinity modulates the cellular distribution of Vpu. Taken together, the E28/L33 residues are a determinant for interaction with BST2, which in turn regulates both the cellular distribution and functional properties of the protein.

### The E28/L33 residues are important for CD4 binding and degradation

Efficient interaction between Vpu and BST2 is dependent on both the identities of the amino acids and the proper structures of their respective TMD helices [9, 17, 32]. To ensure access to the BST2 TMD, the Vpu TMD A_10_xxxA_14_xxxA_18_xxxW_22_ interacting interface orients such that it aligns with the bulky residues on the BST2 TMD, facing the opposite direction of the Vpu cytoplasmic domain [17, 32]. Moreover, the TMDs of both proteins have to adopt optimal tilt angles that are permissive to a direct interaction [17, 33]. Along this line, correlations between the extent of Vpu binding and tilt angles have since been reported [33]. As such, our data raise the possibility that the E28A/L33A mutation perturbs the structural orientation of the Vpu TMD. To test this, we evaluated the ability of the E28A/L33A mutant to interact with another host membrane target protein of Vpu, the CD4 viral receptor, which is intercepted in the endoplasmic reticulum (ER) prior to trafficking to more distal cellular compartments and that requires an intact Vpu TMD. Vpu mediates the degradation of CD4 by connecting the viral receptor to components of the ER-associated protein degradation (ERAD) pathway through a process that requires Vpu-CD4 binding via both the TMD and first helix of Vpu, as well as recruitment of the SCF^β-TrCP^ E3 ligase complex [7, 8, 34–36].

**Fig. 7:**
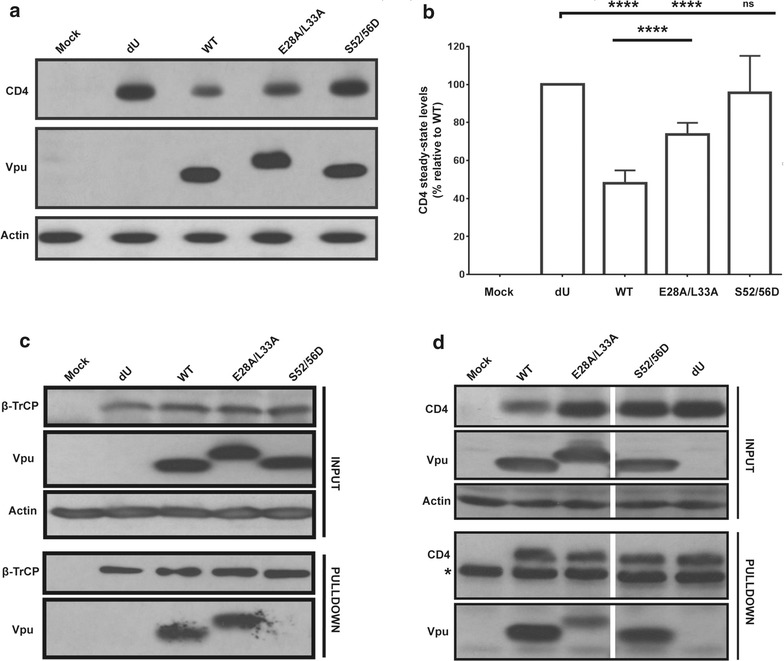
**E28A**/L33A mutation affects the ability of Vpu to target CD4. **a**, **b** E28A/L33A is attenuated for CD4 degradation. HEK293T cells were co-transfected with a proviral construct encoding WT Vpu or the indicated Vpu mutant proviruses and a CD4 expressor, and probed for the steady state levels of CD4. **a** Shown is a representative Western blot. **b** A summary of the densitometric quantifications of the steady-state CD4 levels from independent experiments, together with the SD (n = 7). Statistical analysis was performed using a two-way ANOVA, Tukey’s multiple comparison test. **c** E28A/L33A is efficient at recruiting β-TrCP. HEK293T cells were co-transfected with the indicated proviral constructs and a myc-tagged β-TrCP2 expressor. A Co-IP assay was performed using anti-myc Abs to analyze β-TrCP complexes for the presence of Vpu. **d** E28A/L33A is attenuated for CD4 binding. HEK293T cells were co-transfected as in (**a**), and a Co-IP assay performed using anti-CD4 Abs to pull down Vpu. Shown is a representative Western blot indicating the amount of Vpu in the lysate and pulled down fractions in each case. The *asterisk* denotes an Ab-related band

First, to assess the ability of the Vpu mutants to degrade CD4, HEK293T cells were co-transfected with a CD4 expressor and proviruses expressing WT Vpu or Vpu mutants (dU, E28A/L33A or S52/56D), and CD4 steady-state levels were evaluated by Western blotting. While WT Vpu was efficient at mediating CD4 degradation relative to dU, the S52/56D mutant was significantly impaired for this function, owing to the fact that it is defective for β-TrCP recruitment (Fig. 7a–c). Importantly, the E28A/L33A mutant was significantly attenuated for CD4 degradation, yet retained the ability to efficiently interact with β-TrCP (Fig. 7a–c). We then asked whether the E28A/L33A mutant was still able to bind CD4. Towards this, we co-transfected HEK293T cells with a CD4 expressor and the proviral Vpu mutants, and performed Co-IP assays using anti-CD4 Abs to pull down Vpu. Whereas WT Vpu and S52/56D were efficiently pulled down, the E28A/L33A mutant was not, indicating that the E28/L33 residues contribute to the interaction of Vpu with CD4 (Fig. 7d).

Taken together with the BST2 binding data, the results demonstrate that the E28/L33 residues are essential for optimal binding to both BST2 and CD4.

### E28 is a determinant for both Vpu-mediated CD4 degradation and BST2 antagonism

**Fig. 8:**
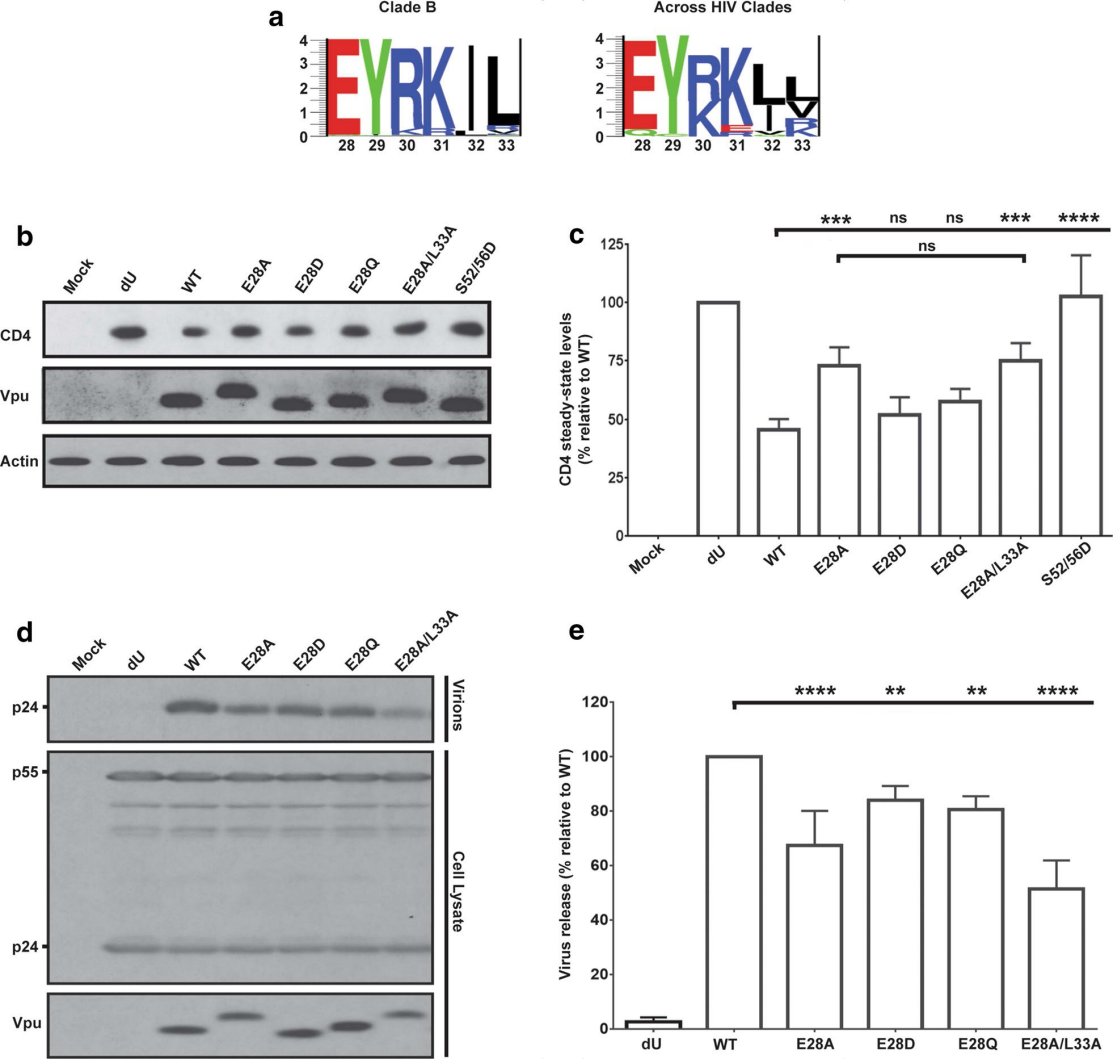
The conserved glutamic acid residue within the hinge region of Vpu modulates the protein functions. **a** Logo plots demonstrating the amino acid conservation at each position within the 28EYRKIL33 transmembrane-proximal hinge region of Vpu isolates from HIV-1 Clade B (*left panel*) as well as from all HIV-1 clades (*right panel*) [49]. **b**, **c** CD4 degradation mediated by E28 single-point mutants. **b** A representative Western Blot indicating steady state levels of CD4 following co-transfections of HEK293T cells with a CD4 expressor and the indicated proviral constructs. **c** Quantitative densitometric analyses of efficiency of Vpu mutants to degrade CD4 from a compilation of five independent experiments. **d**, **e** E28 is important for efficient enhancement of virus release in HeLa cells transfected with proviral constructs encoding Vpu single-point mutations. **d** A representative Western blot showing the amount of virion-associated p24 released into the supernatant (virion) and Gag products (p24 and p55) in the cell lysate. **e** A summary of the efficiency of virus release (quantified and expressed as mentioned in Fig. 4 legend) from five different experiments. For both (**c**) and (**e**), the error bars represent SD. Statistical analyses were performed using a two-way ANOVA, Tukey’s multiple comparison test

We next wanted to delineate the contributions of the E28/L33 residues towards the observed functional and binding phenotypes. Considering the proximity of the E28 residue to the functionally important TMD, as well as the fact that it is highly conserved across Vpu variants (Fig. 8a), we hypothesized that it is primarily the glutamate that acts as a membrane anchor for this TMD region. Focusing on the glutamate, we introduced E28A, E28D and E28Q point mutations in order to discern whether the charge, polarity and/or intrinsic structural elements are essential for targeting CD4 and/or BST2. Noteworthy, the Gln is also found in other variants of Vpu, especially those from HIV-1M G subtype [49].

**Fig. 9:**
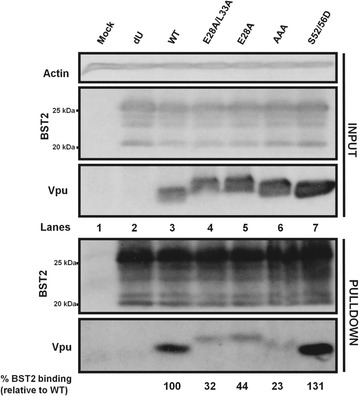
E28 residue is a key modulator of binding of Vpu to BST2. Co-IP following co-transfection of HEK293T cells with a proviral construct encoding WT Vpu, E28A/L33A or E28A Vpu mutants and a BST2 expressor. Below the blot is the extent of BST2 binding of each Vpu mutant, relative to WT Vpu (set at 100%). BST2 binding efficiency was determined as indicated in Fig. 5 legend

In terms of mediating CD4 degradation, our results indicate that whereas both E28D and E28Q mutants were comparable to WT Vpu, the E28A mutant was significantly defective, suggesting that the E28 position can accommodate a polar or semi-conservative residue (Fig. 8b, c). In fact, the E28A mutation was sufficient to account for the difference or attenuated phenotype observed with the E28A/L33A mutant. For BST2 antagonism, however, it was particularly interesting that all the mutants showed a modest, yet statistically significant attenuation compared to WT Vpu (Fig. 8d, e). Noteworthy, while the L33 position was previously reported to be dispensable for virus release [18], mutating it in the context of the E28A was necessary in order to give the defective phenotype observed with the E28A/L33A mutant. Similar to their BST2 antagonistic activities, the single mutants were all attenuated for Vpu-mediated BST2 degradation (Additional file 3: Fig. S3). As such, the data argue that the glutamate residue offers some unique intrinsic properties that are important for targeting BST2. In line with these observations, E28A also displays a reduced binding to BST2 (Fig. 9). Interestingly, this attenuation in BST2 binding appears slightly less than that of the E28A/L33A mutant, suggesting that the intermediate ability of Vpu E28A to counteract BST2 correlates with its extent of binding to the restriction factor. Altogether, the above observations are consistent with a role of the glutamic acid residue in allowing for optimal counteraction of BST2 as well as efficient targeting and degradation of CD4.

## Discussion

In this study, we investigated the contribution of the highly conserved transmembrane-proximal hinge region (28EYRKIL33) towards Vpu-mediated BST2 antagonism. We show that while residues in this region have no intrinsic activity on the cellular distribution of Vpu in the absence of BST2, they regulate the ability of Vpu to bind BST2 and, consequently, govern both BST2-dependent trafficking properties of the protein as well as co-localization with BST2. We further show that these conserved residues are important not only for BST2 counteraction but also for the capacity of Vpu to efficiently target the CD4 viral receptor for degradation in the ER. Ultimately, our results reveal that these conserved hinge region residues are important for the ability of Vpu to optimally counteract BST2 and degrade CD4. While mutagenesis analysis of conserved residues revealed subtle differences regarding the relative importance of these residues for BST2 or CD4 targeting, evidence points towards a potential role of these residues in the maintenance of the Vpu TMD conformational configuration such that interactions with membrane-associated host targets, via association between their respective TMDs, are favoured.

Optimal binding of Vpu to BST2 is a critically important pre-requisite for antagonism. To date, this extensively characterized direct binding has been shown to involve residues within the transmembrane regions of both proteins, with no evidence implicating the Vpu hinge region residues in said interaction [9, 16, 17]. Our finding that the hinge region residues also regulate this interaction was therefore unexpected, and highlights a previously unappreciated important determinant for BST2 binding that occurs beyond the reported TMD residues. This binding modulation is likely due to a regulatory effect on the conformational integrity of the Vpu TMD, as evidenced by the defects in Vpu-mediated functions that are dependent on interactions involving the TMD. Accordingly, these residues are important for targeting BST2 and CD4, and could conceivably play a role in the interaction with other membrane-associated host targets of Vpu including NTB-A, PVR, SNAT1 and CCR7. Preservation of both secondary and tertiary structures, as well as positioning of the TMD likely enable interactions with these target proteins. The conformational integrity of the Vpu TMD is indeed essential for optimal biological activity, as recently demonstrated by the positive correlation between alterations in tilt angles and attenuation in BST2 antagonism observed in Vpu TMD mutants [33]. Similarly, altered tilt angles of BST2 TMD mutants confined within lipid bilayers desensitized them to the antagonistic activity of Vpu, yet they retained an intrinsic ability to interact with Vpu in solution [17]. Furthermore, nuclear magnetic resonance data on the direct helix–helix interaction between Vpu and BST2 TMDs demonstrate the need for appropriate alignment of the Vpu interacting interface in order to directly contact the bulky BST2 TMD residues that likely fit between the alanine-based interface of the Vpu TMD [17]. Consistently, the Vpu TMD interface faces away from the cytoplasmic domain of the protein, ensuring accessibility to its binding partners [32].

The evidence presented herein implying a contribution of the Vpu hinge region residues, especially the glutamate at the TMD-cytoplasmic interface, towards anchoring the TMD is in accordance with previous findings demonstrating a modulatory function of a similarly positioned glutamate residue in the conformational positioning of a model poly-Leu TMD helix [37]. Mutating the glutamate residue is believed to abrogate its role of linking the TMD hydrophobic segment to the aqueous phase, resulting in a mismatch between the hydrophobic helix relative to the length of phospholipids in the bilayer and, ultimately, altered TMD tilt angles [38, 39]. Interestingly, glutamate residues flanking TMDs of bacteriorhodopsin were also shown to modulate the conformational configurations of the TMDs, including the mobility of Ala and Val TMD residues [40, 41]. More interesting still, and corroborating our findings herein, these studies also document the uniqueness of the glutamate intrinsic properties, as it could not be substituted even with a semi-conservative glutamine residue, [40, 41]. This likely reflects the superior capability of the Vpu Glu to form stabilizing interactions with the positive residues R30/K31/R34 via hydrogen bonds or salt bridges [42, 43].

The interaction of Vpu and BST2 mediates the recruitment of clathrin AP complexes that govern BST2 intracellular sequestration, degradation, endocytosis and displacement from viral assembly sites [21, 22]. Thus, by disrupting Vpu interaction with BST2, mutations of conserved residues of the hinge region are likely to affect the recruitment of AP via the di-leucine trafficking motif in the second helix of Vpu [22]. Indeed, the aberrant BST2-dependent cellular distribution of the E28A/L33A mutant is likely a consequence of such a defect, in line with the notion that mutations that affect AP recruitment lead to defects in cellular localization only in the context of the Vpu–BST2 complex [20, 22]. In fact, the extent of BST2 counteraction by the E28A/L33A mutant appears to correlate with both its significantly compromised binding phenotype and its slightly altered cellular localization in the presence of BST2. Interestingly, the altered Vpu localization triggered by the E28A/L33A mutant was not found to be additive with the localization alteration induced by the second helix di-leucine mutant, consistent with the requirement of a Vpu–BST2 physical interaction for recruitment of AP proteins [20, 22]. Thus, our data support previous results emphasizing the strong correlation between Vpu-mediated BST2 antagonism and both Vpu–BST2 interaction and Vpu–BST2 complex cellular localization.

The functional contribution of the glutamate residue is distinct from that mediated by the other hinge region residues (28EYRKIL33). While our previous findings showed that Y29 was dispensable for BST2 antagonism, the R30/K31 residues were found important, yet were dispensable for both binding to BST2 and CD4 degradation [18]. Mechanistically, unlike the E28/L33 residues, the R30/K31 residues appear important for the intrinsic cellular distribution of Vpu within the TGN [18], implying that they are unable to efficiently intercept recycling and/or newly synthesized BST2. Altogether, therefore, these E28/R30/K31/L33 flexible hinge region residues play unique, multifaceted functional roles, much like the other membrane-distal flexible linker occurring between the two alpha helices of Vpu that regulates binding to both AP proteins and β-TrCP [6, 7, 22].

Considering the functional importance of the transmembrane-proximal hinge region glutamate, it is not surprising that this residue is highly conserved across HIV-1M Vpu variants. Consistent with our findings herein, a recent study reported that only two out of a pool of 304 patient-derived Vpu alleles had a lysine substitution at this position, and were significantly impaired in their ability to mediate both BST2 antagonism and surface CD4 downregulation [44]. Intriguingly, in the case of Vpu variants with a canonical di-leucine motif within the second helix, such as in VpuB, the hinge region residues offer some plasticity in that only a single amino acid substitution would be sufficient to generate an optimal trafficking motif (ExxxIL vs ExxxLL). Along this line, analysis of the Los Alamos HIV database reveals that 13% of VpuB variants (18) with mutations at critical positions within their helix-2 di-leucine motifs (136 out of a total of 1648 Vpu variants) have optimal putative transmembrane-proximal hinge region trafficking signals [49]. Indeed, while the influence of the hinge region residues on BST2 binding, and hence Vpu (NL4.3) cellular distribution, is consequential, we cannot formally rule out that these residues, which are part of a putative acidic di-leucine trafficking motif, also contribute directly to Vpu–BST2 complex trafficking. Future studies aimed at characterizing the exact compartments into which the mislocalized Vpu (NL4.3) is distributed would be essential in discerning whether these residues play any role in modulating trafficking of the protein in the presence of BST2. Nevertheless, there appears to be a selective pressure to maintain the glutamate residue at this position in order to ensure optimal activity of the TMD as shown herein. Of note, in the case of VpuC, the hinge region residues do meet the canonical sequence requirements for a di-leucine motif (ExxxLL), whereas the second helix possesses a putatively sub-optimal di-leucine motif (ExxxMV). It remains to be elucidated whether the hinge region glutamate residue in VpuC contributes towards binding to BST2 and/or CD4, and whether it is involved as a part of an active trafficking signal.

## Conclusions

Overall, this work demonstrates an important regulatory role of the Vpu transmembrane-proximal hinge region for the ability of the protein to optimally interact with its target host factors. As such, it is an important determinant for optimal Vpu-mediated BST2 antagonism and CD4 degradation, both of which are key for viral pathogenesis. Furthermore, considering that this domain is highly conserved, occurs within the cytosolic region of the protein, and modulates binding to various host targets, it may represent an attractive target for the development of anti-Vpu inhibitors, unlike the TMD, which by virtue of its association to the membrane is less accessible.

## Methods

### Antibodies and reagents

Rabbit sera for pre-immune and anti-Vpu, mouse anti-p24 monoclonal and anti-CD4 (OKT4) Abs were described previously [18]. Rabbit serum for anti-BST2 and mouse anti-myc (clone 9E10) Abs used for co-immunoprecipitation were also described previously [14]. Polyclonal sheep anti-TGN46 and mouse anti-BST2 used in confocal microscopy immunostainings were obtained from Serotec and Abnova, respectively. Rabbit Abs directed against CD4 (Santa Cruz Biotechnology), Myc (Sigma) and actin (Sigma) used for Western blot analyses were obtained from commercial sources as indicated. All secondary Alexa-conjugated immunoglobulin G (IgG) Abs used for flow cytometry and confocal microscopy were obtained from Life Technologies. Paraformaldehyde (PFA) was obtained from Sigma.

### Plasmids, cell lines and transfections

#### Plasmids

WT HIV-1 NL4.3 was obtained through the NIH AIDS Reagent Program and a dU provirus plasmid was kindly provided by Dr. Klaus Strebel. All Vpu mutants described were in the parental NL4.3 provirus genome background, and were generated by PCR-based Quick-change site-directed mutagenesis according to standard protocols using cloned Pfu (Agilent). The oligonucleotides used are (only the sense strand is shown): ***E28A*** 5ʹ-CATAGTAATCATAGCATATAGGAAAATATTAAGAC-3ʹ; ***E28D*** 5ʹ-CCATAGTAATCATAGATTATAGGAAAATATTAAGAC-3ʹ; ***E28Q*** 5ʹ- CCATAGTAATCATACAATATAGGAAAATATTAAGAC-3ʹ; ***E28A/L33A*** 5ʹ-GTCCATAGTAATCATAGCATATAGGAAAATAGCAAGACAAAGAAAAATAGACAG-3ʹ; ***E59K/L63F*** 5ʹ-CATAGAATATAGGAAATTATTAAGACAAAGAAAAATAGACAGG-3ʹ; ***S52/56D*** 5ʹ-GAAAGAGCAGAAGACGATGGCAATGAGGATGAAGGAGAAGTATCAGCA-3ʹ; ***A14L*** 5ʹ- GTAGCATTAGTAGTATTAATAATAATAGCAATAGTTGTGTGGTCC-3ʹ; ***A18L*** 5ʹ- GCATTAGTAGTAGCAATAATAATATTAATAGTTGTGTGGTCCATAG-3ʹ; ***A10L/A18L*** 5ʹ-CCTATAATAGTAGCAATAGTACTATTAGTAGTAGCAATAATAATACTAATAGTTGTGTGGTCC-3ʹ; ***A10L/A14L/A18L*** 5ʹ-CCTATAATAGTAGCAATAGTATTATTAGTAGTATTAATAATAATATTAATAGTTGTGTGGTCC-3ʹ. For the E28A/L33A-E59K/L63F mutant, sequential mutagenesis was performed using the E59K/L63F oligonucletides on the E28A/L33A template plasmid. The provirus Vpu-AAA mutant was also described previously [45], except that the provirus backbone used here did not encode GFP. All plasmid constructs were confirmed by DNA sequencing. The expression plasmids pCR3.1, encoding the short isoform of BST2 [45], SVCMV-CD4 [46], and pSVCMV VSV-G encoding the vesicular stomatitis virus glycoprotein G (VSV-G) [14] were described previously as indicated. The expression plasmid for pCR3.1 HA-BST2 gene was kindly provided by Dr. Bieniasz [47] while the pcDNA/Myc-His-β-TrCP plasmid was obtained from Dr. Richard Benarous [7].

**Cell lines** HEK293T and HeLa cells were obtained from the American Type Culture Collection. Both cell types were maintained in Dulbecco’s Modified Eagle Medium (DMEM, Wisent) supplemented with 10% fetal bovine serum (FBS) and a combination of Penicillin–Streptomycin antibiotics. HeLa cells depleted of BST2 were generated by transducing lentiviral vector particles encoding shRNA targeting BST2 [48] or, as a control, a non-targeting shRNA. The HeLa-TZM-bl cells were obtained through the NIH AIDS Reagent Program.

**Transfections** HEK293T and HeLa cells were seeded overnight and transfected using the calcium-phosphate method and lipofectamine 2000™ (Invitrogen), respectively.

### Virus particle release assay

Viral particle release was analyzed by Western blot as described previously [14]. The intensity of Gag signal was measured by scanning densitometry analyses using ImageJ software (NIH). The ratio of virion-associated Gag (p24) signal to cell-associated Gag (p24 and p55) was indicative of the viral particle release efficiency. Histograms indicate Vpu-specific virus release efficiencies following subtraction of any background or non-Vpu specific effect as determined by the dU control. Vpu-mediated virus release efficiency was normalized to the value obtained from HIV-1 WT Vpu cultures, which was arbitrarily set at 100%.

### BST2 surface staining and flow cytometry

BST2 cell-surface staining was performed on HeLa cells co-transfected with proviral constructs expressing the indicated mutants together with a GFP expressor plasmid for gating purposes. Preparation of cells and flow cytometry analysis were described previously [14]. Surface BST2 down-regulation efficiency was determined by subtracting BST2 geometric mean fluorescence intensity (MFI) values obtained from GFP-expressing (transfected cells) cells from BST2 MFI values obtained from GFP non-expressing cells. For histograms, the efficiency of BST2 downregulation was expressed relative to that obtained from HIV-1 WT Vpu cultures, which was arbitrarily set at 100%.

### Production of VSV-G pseudotyped HIV-1 viruses

HEK293T cells were co-transfected with NL4.3 (or appropriate mutants) proviral constructs and pSVCMV VSV-G as described previously [15]. Forty-eight hours post-transfection, supernatants of transfected cells were clarified by centrifugation, filtered through a 45-μm filter, and pelleted by ultracentrifugation onto a 20% sucrose-PBS cushion for 2 h at 112,000×*g* at 4 °C. Concentrated viruses were resuspended in DMEM supplemented with 10% FBS. Viruses were titrated using a standard MAGI assay as previously described [14].

### Protein steady-state levels

#### BST2 degradation

HeLa cells were infected with VSV-G-pseudotyped HIV-1 proviruses expressing WT, dU or Vpu mutants at an MOI of 1.5 in presence of polybrene. The infection medium was replaced four hours post-infection. Forty-eight hours post-infection, cell lysates were harvested and lysed in RIPA-DOC buffer. BST2 steady state levels were analyzed by Western blotting as described [14].

#### CD4 depletion

HEK293T cells were co-transfected with a CD4 expressor and the appropriate proviral plasmids. Following lysis at forty-eight hours post-transfection, lysates were subjected to Western blotting and probed for steady-state levels of CD4.

### Cellular localization and confocal microscopy

Cover-slip seeded BST2-expressing or BST2-depleted HeLa cells were transfected with NL4.3 proviral plasmids expressing either WT Vpu or the indicated Vpu mutants. Twenty-four hours post-transfection, cells were fixed for 30 min in 4% PFA and then permeabilized in 0.2% Triton for 5 min. Following washes, permeabilized cells were incubated for 2 h at 37 °C in 5% milk-PBS containing rabbit anti-Vpu, sheep anti-TGN46 and/or mouse anti-BST2 Abs as appropriate. The cells were then washed and incubated with the appropriate Alexa Fluor-coupled secondary Abs for 45 min at room temperature followed by an incubation with 4′,6-diamidino-2-phenylindole (DAPI) for 5 min also at room temperature. All analyses were acquired using a 63× Plan Apochromat oil immersion objective on an LSM710 Observer Z1 laser scanning confocal microscope (Zeiss). Quantitative analyses were performed using the Volocity software (PerkinElmer Inc.), using automated signal thresholding.

### Co-IP binding assays

#### Vpu and BST2 interaction

HEK293T cells were co-transfected with a BST2 expressor plasmid (WT or a short isoform of BST2) together with proviral plasmids expressing either WT Vpu or indicated mutants. Forty-eight hours post-transfection, cells were harvested in PBS/EDTA, centrifuged and lysed in CHAPS buffer (50 mM Tris, 100 mM NaCl, 0.5% CHAPS, pH 7.2) supplemented with a cocktail of protease inhibitors (Protease Inhibitors Complete, with EDTA). An aliquot of 10% of cell lysate was preserved for Western blot loading as “Input” control. Cell lysates were then pre-cleared with protein A Sepharose beads coated with pre-immune rabbit serum for 1 h at 4 °C. Following pre-clearing, the lysates were incubated with rabbit anti-BST2 specific serum for 3 h at 4 °C, and then precipitated using protein A Sepharose beads in a further 3-h incubation at 4 °C. Western blotting was then used to probe for presence of Vpu and BST2 in immunoprecipitated fractions.

#### Vpu and CD4 interaction

HEK293T cells were co-transfected with a CD4 expressing plasmid and the appropriate proviral plasmids, and similar steps were followed as above, except pre-immune mouse serum was used for pre-clearing, and mouse anti-CD4 Abs were used to pull down Vpu.

#### Vpu and β-TrCP binding

HEK293T cells were co-transfected with a myc-tagged β-TrCP2 expressing plasmid and the appropriate proviral plasmids, and similar Co-IP steps were followed as above, except pre-immune mouse serum was used for pre-clearing, and mouse anti-myc Abs were used to pull down Vpu.

### Statistical analysis

Statistical analyses for confocal microscopy data were performed using an unpaired, Mann–Whitney test. All other analyses were performed using two-way ANOVA, with Tukey’s multiple comparison test. Values were considered statistically significant at ‘p’ values of <0.05. For all statistical analyses, ****, ***, **, * and ‘ns’ denote p < 0.0001, p < 0.001, p < 0.01, p < 0.05 and p > 0.05, respectively (ns = not significant).

## Additional files

**Additional file 1: Figure S1.** Ability of Vpu mutants to mediate BST2 degradation. Shown is a representative Western blot indicating the steady state levels of BST2 in mock-infected HeLa cells (lane 1) as well as in Hela cells following infections with VSV-G-pseudotyped HIV-1 viruses encoding WT Vpu and the indicated Vpu mutants (lanes 2–7). Analysis of BST2 expression in BST2-depleted HeLa cells is also shown in lane 8. Below the blot is the extent of BST2 expression based on densitometric analyses of the intensities of BST2-related band signals obtained from each Vpu mutant, relative to dU (set at 100%).

**Additional file 2: Figure S2.** BST2 binding capacity of Vpu TMD mutants. Co-IP following co-transfection of HEK293T cells with a proviral construct encoding WT Vpu, E28A/L33A or the indicated Vpu TMD mutants and a BST2 expressor. Below each blot is the extent of BST2 binding of each Vpu mutant, relative to WT Vpu (set at 100%). For each condition, BST2 binding efficiency was determined from the ratio obtained from densitometric analyses of the intensities of Vpu- and BST2-related band signals in the immunoprecipitated fractions. The asterisk denotes an Ab-related band.

**Additional file 3: Figure S3.** E28 is important for efficient degradation of BST2. Shown is a representative Western blot indicating the steady state levels of BST2 in mock-infected HeLa cells (lane 1) as well as in Hela cells following infections with VSV-G-pseudotyped HIV-1 viruses encoding WT Vpu and the indicated Vpu mutants (lanes 2–7). Below the blot is the extent of BST2 expression based on densitometric analyses of the intensities of BST2-related band signals obtained from each Vpu mutant, relative to dU (set at 100%).
