# Supplementary figures and images for: Conserved residues within the HIV-1 Vpu transmembrane-proximal hinge region modulate BST2 binding and antagonism
Source: Retrovirology. 2017 Mar 14;14:18. doi: 10.1186/s12977-017-0345-6 (PMC5348903; doi:10.1186/s12977-017-0345-6)

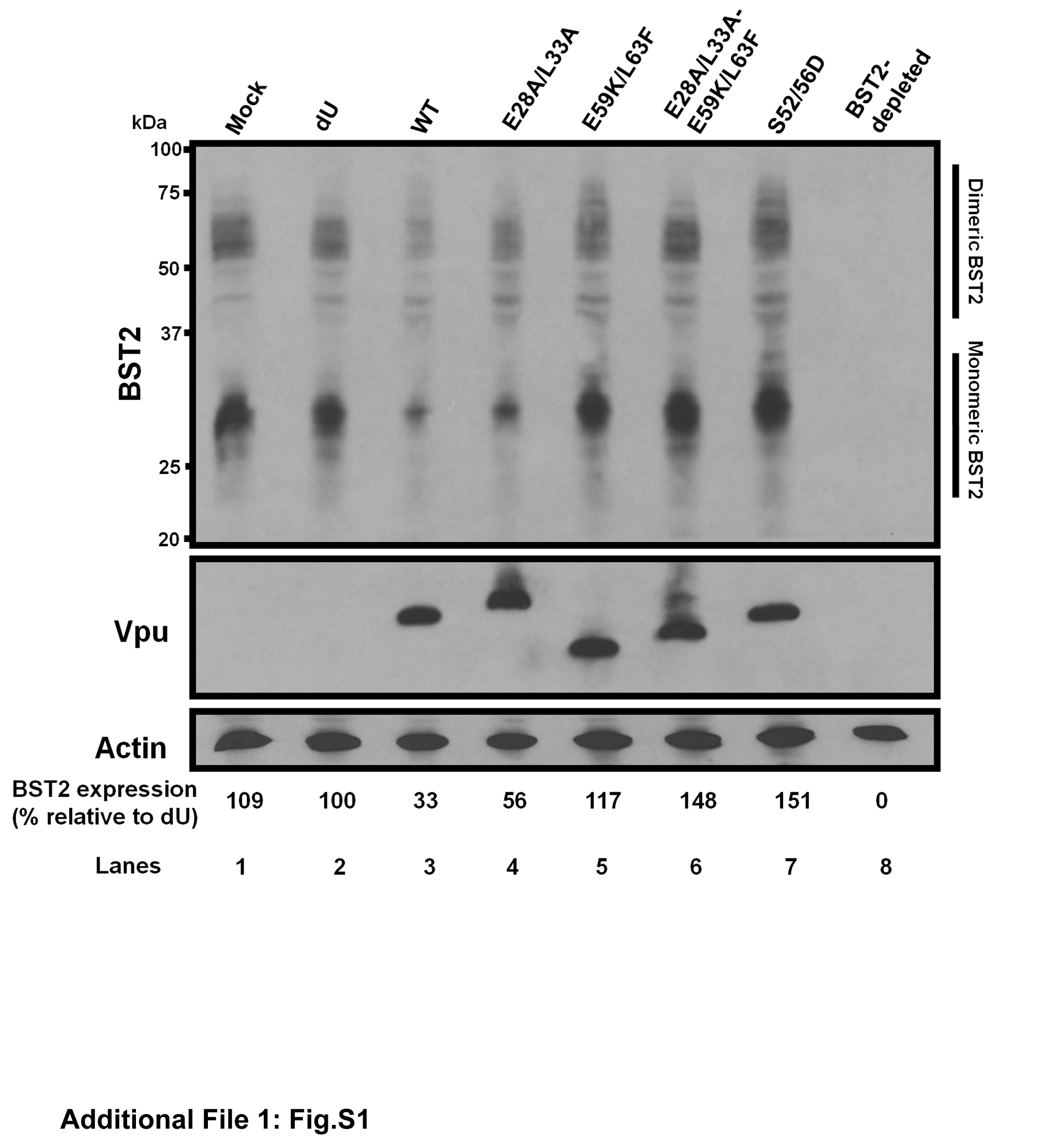

Supplement: Supplementary file 1 — Additional file 1: Figure S1. Ability of Vpu mutants to mediate BST2 degradation. Shown is a representative Western blot indicating the steady state levels of BST2 in mock-infected HeLa cells (lane 1) as well as in Hela cells following infections with VSV-G-pseudotyped HIV-1 viruses encoding WT Vpu and the indicated Vpu mutants (lanes 2–7). Analysis of BST2 expression in BST2-depleted HeLa cells is also shown in lane 8. Below the blot is the extent of BST2 expression based on densitometric analyses of the intensities of BST2-related band signals obtained from each Vpu mutant, relative to dU (set at 100%). [file 12977_2017_345_MOESM1_ESM.tif]

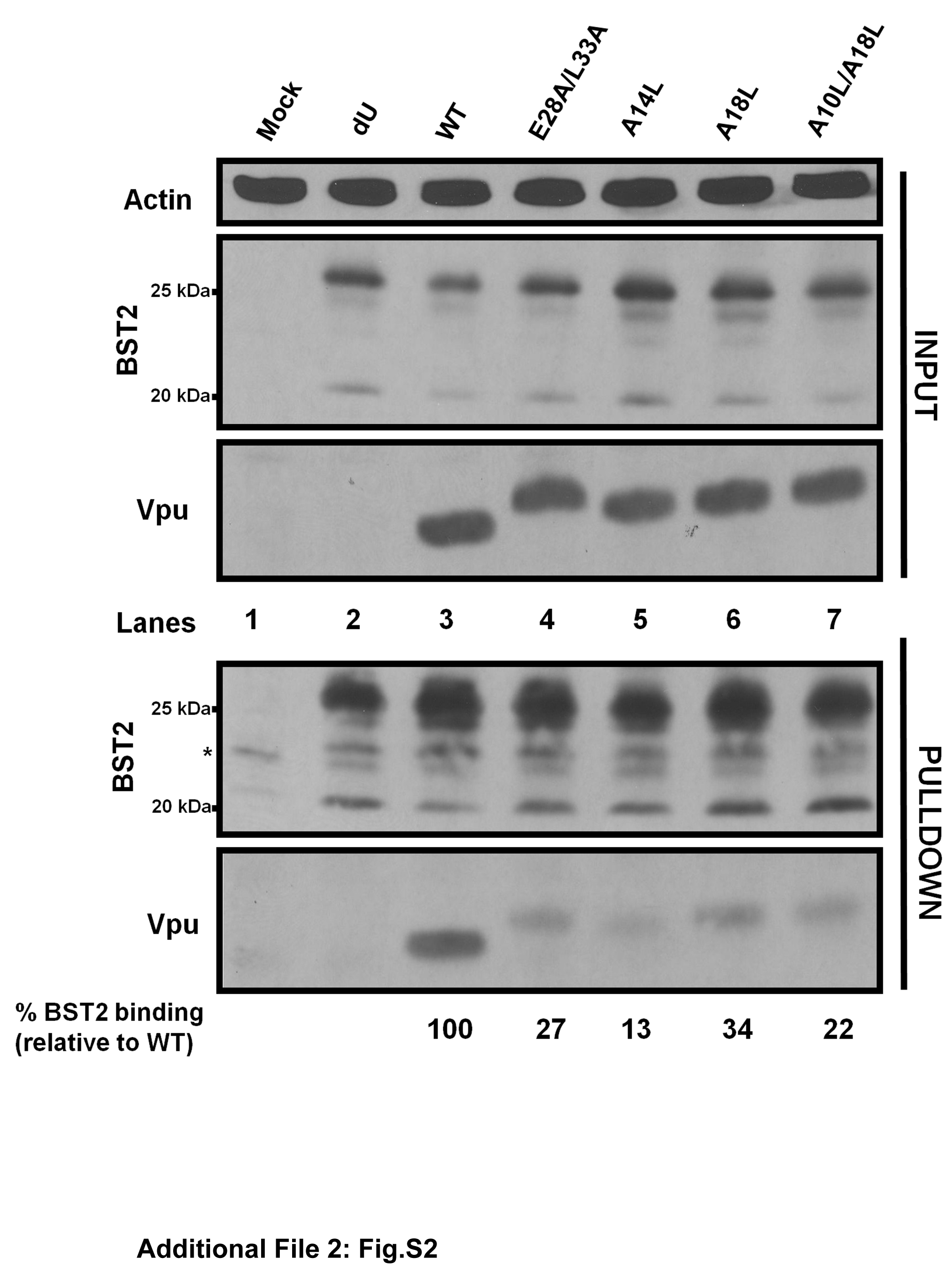

Supplement: Supplementary file 2 — Additional file 2: Figure S2. BST2 binding capacity of Vpu TMD mutants. Co-IP following co-transfection of HEK293T cells with a proviral construct encoding WT Vpu, E28A/L33A or the indicated Vpu TMD mutants and a BST2 expressor. Below each blot is the extent of BST2 binding of each Vpu mutant, relative to WT Vpu (set at 100%). For each condition, BST2 binding efficiency was determined from the ratio obtained from densitometric analyses of the intensities of Vpu- and BST2-related band signals in the immunoprecipitated fractions. The asterisk denotes an Ab-related band. [file 12977_2017_345_MOESM2_ESM.tif]

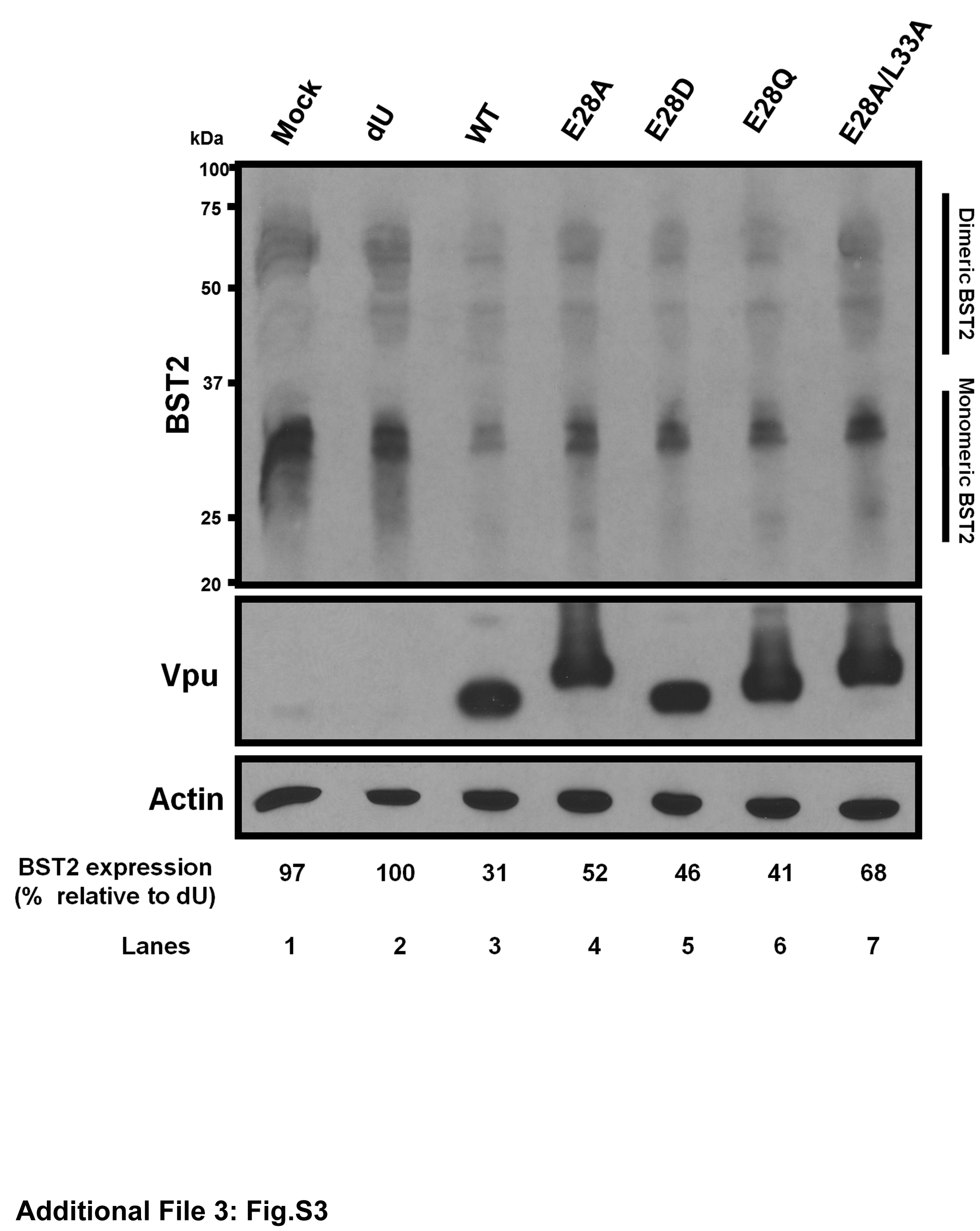

Supplement: Supplementary file 3 — Additional file 3: Figure S3. E28 is important for efficient degradation of BST2. Shown is a representative Western blot indicating the steady state levels of BST2 in mock-infected HeLa cells (lane 1) as well as in Hela cells following infections with VSV-G-pseudotyped HIV-1 viruses encoding WT Vpu and the indicated Vpu mutants (lanes 2–7). Below the blot is the extent of BST2 expression based on densitometric analyses of the intensities of BST2-related band signals obtained from each Vpu mutant, relative to dU (set at 100%). [file 12977_2017_345_MOESM3_ESM.tif]
